# The Effects of Acute and Chronic Selective Phosphodiesterase 1 Inhibition on Smooth Muscle Cell-Associated Aging Features

**DOI:** 10.3389/fphar.2021.818355

**Published:** 2022-01-31

**Authors:** Keivan Golshiri, Ehsan Ataei Ataabadi, Annika A. Jüttner, Gretchen L. Snyder, Robert E Davis, Amy Lin, Lei Zhang, René de Vries, Ingrid M Garrelds, Frank P. J. Leijten, A. H. Jan Danser, Anton J. M. Roks

**Affiliations:** ^1^ Department of Internal Medicine, Erasmus Medical Center, Rotterdam, Netherlands; ^2^ Intra-Cellular Therapies, Inc., New York, NY, United States

**Keywords:** phosphodiesterase, vascular aging, smooth muscle cell, cardiovascular, NO-cGMP pathway, inflammation

## Abstract

Age-related cardiovascular diseases (CVDs) remain among the leading global causes of death, and vascular smooth muscle cell (VSMC) remodeling plays an essential role in its pathology. Reduced NO-cGMP pathway signaling is a major feature and pathogenic mechanism underlying vasodilator dysfunction. Recently, we identified phosphodiesterase (PDE) 1, an enzyme that hydrolyzes and inactivates the cyclic nucleotides cAMP and cGMP, and thereby provides a potential treatment target for restoring age-related vascular dysfunction due to aging of VSMC. Based on this hypothesis, we here tested the effects of PDE1 inhibition in a model of SMC-specific accelerated aging mice. SMC-KO and their WT littermates received either vehicle or the PDE1 inhibitor lenrispodun for 8 weeks. Vascular function was measured both *in vivo* (Laser Doppler technique) and *ex vivo* (organ bath). Moreover, we deployed UV irradiation in cell culture experiments to model accelerated aging in an *in vitro* situation. SMC-KO mice display a pronounced loss of vasodilator function in the isolated aorta, the cutaneous microvasculature, and mesenteric arteries. *Ex vivo*, in isolated vascular tissue, we found that PDE1 inhibition with lenrispodun improves vasodilation, while no improvement was observed in isolated aorta taken from mice after chronic treatment *in vivo*. However, during lenrispodun treatment *in vivo*, an enhanced microvascular response in association with upregulated cGMP levels was seen. Further, chronic lenrispodun treatment decreased TNF-α and IL-10 plasma levels while the elevated level of IL-6 in SMC-KO mice remained unchanged after treatment. PDE1 and senescence markers, *p16* and *p21*, were increased in both SMC-KO aorta and cultured human VSMC in which DNA was damaged by ultraviolet irradiation. This increase was lowered by chronic lenrispodun. In contrast, lenrispodun increased the level of PDE1A in both situations. In conclusion, we demonstrated that PDE1 inhibition may be therapeutically useful in reversing aspects of age-related VSMC dysfunction by potentiating NO-cGMP signaling, preserving microvascular function, and decreasing senescence. Yet, after chronic treatment, the effects of PDE1 inhibition might be counteracted by the interplay between differential PDE1A and C expression. These results warrant further pharmacodynamic profiling of PDE enzyme regulation during chronic PDE1 inhibitor treatment.

## Introduction

Age-related cardiovascular diseases (CVDs) remain among the leading global causes of death, and thus the relationship between aging and cardiovascular pathology is an important health care issue (Lakatta and Levy, 2003; Fajemiroye et al., 2018). Vascular smooth muscle cell (VSMC) remodeling plays an essential role in cardiovascular diseases. It is characterized by diminished endothelium–dependent and–independent vasodilation, increased stiffness, extracellular matrix deposition, and a thickened intima consisting of infiltrating VSMCs. Further increases inflammatory cells resulting in local inflammation are also seen (Ferrucci and Fabbri, 2018; Jaminon et al., 2019; Steven et al., 2019). Together these changes result in a broadly dysfunctional vasculature (Sprague and Khalil, 2009; Fajemiroye et al., 2018; Steven et al., 2019; Del Campo et al., 2020; Tyrrell and Goldstein, 2021). Impaired vasodilation and the overproduction of inflammatory markers are considered as trigger factors of CVD onset (Sprague and Khalil, 2009; Widmer and Lerman, 2014; Fajemiroye et al., 2018; Steven et al., 2019). The identification of a pharmacological target that can be used to mitigate these diverse processes would be expected to be of great value.

The presence of functional vascular endothelium is essential for acetylcholine (ACh)-mediated vasodilation and it is well established that nitric oxide (NO) released from the endothelium is mainly responsible for the relaxation induced by ACh. Therefore, there is also an option to circumvent the endothelium and stimulate the vascular smooth muscle cells, directly, by the mean of NO donors such as sodium nitroprusside (SNP) (Knox et al., 2019). We have previously identified phosphodiesterase 1, an enzyme that metabolizes the cyclic nucleotides cAMP and cGMP, as a potential target for pharmacological intervention. Both cAMP and cGMP are important second messengers that regulate numerous signaling pathways and play a vital role in aging-related CVD (Lehners et al., 2018). Cyclic AMP is synthesized by adenylyl cyclases (ACs), and cGMP is produced from both the cytosolic soluble (sGC) and membrane-bound particulate guanylyl cyclases (pGC) (Carvalho et al., 2021). Activation of ACs and GCs in VSMCs occurs upon binding a myriad of different naturally occurring agonists to their receptors, of which respectively vasodilating prostaglandins (AC) and natriuretic peptides (pGC) are exemplary. Nitric oxide is the main stimulator of sGC, binding directly to a heme moiety. Increases in the levels of cAMP and cGMP lead to vasorelaxation and a decrease of vascular hypertrophy and are therefore believed to protect against hypoperfusion, hypertension, and hypertrophic vascular remodeling, three main hallmarks of aging. Phosphodiesterases are enzymes that provide homeostatic control of cyclic nucleotide levels through their conversion to the non-cyclic form. Of more than 100 PDE subtypes, PDE1 plays a vital role in vasculature. It consists of three distinct isomers, PDE1A, PDE1B, and PDE1C and regulates VSMC growth and relaxation by adjusting the cyclic nucleotide levels in confined intracellular microdomains of the VSMC. However, the effects of PDE1 inhibition as an intervention has not yet been explored in the aging vasculature (Golshiri et al., 2020a). Many studies show that PDEs are regulated in response to changes in (patho)physiological conditions (Yanaka et al., 2003; Bautista Niño et al., 2015; Knight et al., 2016; Khammy et al., 2017; Hashimoto et al., 2018; Zhang et al., 2019; Golshiri et al., 2020b). Relevant for vascular structural remodeling, PDE1 can become the predominant PDE during VSMC phenotype transition from the synthetic to the proliferative/migratory state putatively affecting the pathological remodeling of the vascular wall. However, this potential role needs to be examined *in vivo* (Zhang et al., 2019). Our previous work applying acute *ex vivo* tests with PDE1 inhibitors suggested that PDE1 might underlie the development of vasomotor dysfunction during aging (Bautista Niño et al., 2015). Our recent examination of the effects of acute and chronic PDE1 inhibition in a mouse model of accelerated aging showed an acute contribution to vasodilation specifically in aged mice, and protection against aging-related vasomotor dysfunction after chronic treatment (Golshiri et al., 2021). It is unclear however if this effect is mediated through a specific effect on smooth muscle cells.

To test such an effect, we have recently generated a mouse model of accelerated VSMC aging based on the principle that DNA damage is a causal factor in aging (Ataabadi et al., 2021; Yousefzadeh et al., 2021a). Humans harbouring DNA repair defects show accelerated aging, and this can be mimicked in mice (Vermeij et al., 2016). Mice that lack certain vital components of the DNA repair system, for instance, excision repair cross-complementation group 1 (ERCC1), a versatile endonuclease that is involved in several DNA repair systems, can display a broad set of progressive aging features shortly after birth, culminating in premature death in a period of several weeks to months (Durik et al., 2012; Bautista-Niño et al., 2016; Vermeij et al., 2016; Wu et al., 2017; Bautista-Niño et al., 2020). Such models can be used for convenient testing of aging effects of interventions. We have previously shown this possibility in *Ercc1* ^*Δ/-*^ mice, which lack proper ERCC1 function in all cells of the body (Wu et al., 2017; Faridounnia et al., 2018; Golshiri et al., 2020a; Bautista-Niño et al., 2020). To study the isolated effect in VSMC aging, we have recently generated and characterized SM22α^Cre+^—*Ercc1flox* (SMC-KO) hybrid mice. These SMC-KO display many human-like vascular aging features starting from the age of about 3 months, including increased vascular wall thickness, stiffness, and diminished macro-and microvascular relaxation that is mainly explained by reduced NO-cGMP pathway signaling (Golshiri et al., 2020a; Ataabadi et al., 2020). The development of a new highly selective PDE1 inhibitor, lenrispodun, provides the opportunity to characterize the functional role of PDE1 in controlling vascular aging properties (Snyder et al., 2016). In the present study, we investigated the role of PDE1 in smooth muscle cell-specific aging features in SMC-KO mice, both *in vivo* and *in vitro,* with the main focus on changes in NO-cGMP responsiveness.

## Material and Methods

### Animals

SM22α^Cre+^ *Ercc1* ^fl/−^ (SMC-KO) and SM22α^Cre+^ *Ercc1* ^fl/+^ littermate (SMC-LM) F1 mice with a hybrid C57BL6J:FVB background were generated by cross-breeding of B6.129S6-Tagln^tm2(cre)Yec/J^ (The Jackson Laboratory, United States) and Ercc1^tm2Dwm^ (institutional colony Erasmus MC) parents that were derived from cross breedings for >10 generations with wild-type mice of a pure C57BL6J and FVB background, respectively. This hybrid construction was used to avoid strain-specific effects of genetic modification. All breeding and animal procedures were performed at the Erasmus MC facility for animal experiments followed by the guidelines from Directive 2010/63/EU of the European Parliament on the protection of animals used for scientific purposes. Mice were housed in individually ventilated cages in a controlled environment (20–22°C, 12 h light:12 h dark cycle) and fed normal chow and water *ad libitum*. The animals were weighed and visually inspected every day to score any visible discomfort and warrant their well-being. All animal studies were performed in accordance with the Principles of Laboratory Animal Care and with the guidelines approved by the independent Dutch Animal Ethical Committee.

### Study Design

In total, 22 SMC-KO mice, which have low *Ercc1* expression in the aorta, and 22 SMC-LM, which have a normal intact *Ercc1* expression, were randomized into four groups at the age of 10 weeks: one group of SMC-KO mice and one of the SMC-LM were given unadulterated drinking water; another group of SMC-KO and SMC-LM mice received the PDE1 inhibitor lenrispodun (40 mg/kg/day) via drinking water for 12 weeks (Snyder et al., 2016; Hashimoto et al., 2018). Both male and female animals were used. Superficial blood flow was measured 1 week prior to sacrifice.

### Analysis of Plasma Cytokine Levels

Plasma protein levels of IL-1β, IL-2, IL-6, IL-10, TNF-α, and IFN-γ were measured using a V-Plex Meso Scale Discovery (MSD) Multiplex spot assay Mouse Neuroinflammation 1 panel. All samples were diluted at a ratio of 1:4 with diluent 41—provided in the MSD kit. Samples and Standards were run in triplicates according to manufacturer instructions and analyzed with MSD Discovery Workbench software (Meso Scale Discovery, Gaithersburg, MD).

### cGMP Content

The concentration of cGMP in the urine was measured using the Cayman Cyclic GMP ELISA kit. Urine samples were diluted 1:7,500 in ELISA buffer and assayed directly following the manufacturer’s instructions. Values of the samples were interpolated from the standard curve using GraphPad Prism software.

### Cell Culture and UV Irradiation

To confirm the findings in our animal model, the effects of aging on human VSMC in culture were evaluated. The cells were obtained from normal medial aortic explants from 5 donors and cultured in SmGM™-2 BulletKit™. The medium was replaced every 48 h. All experiments were performed using cells at passage 8. Prior to UV irradiation, cells were seeded into 6-well culture plates and allowed to reach ∼50% confluency. Cells were rinsed twice with 1x Phosphate-Buffered Saline (PBS, in 500 ml: 0.0168 mol monobasic sodium phosphate, 0.072 mol dibasic sodium phosphate, 4.5 g NaCl) prior to UV exposures. Plates were uncovered and exposed to UV-C irradiation (10 J ⁄ m^2^) while covered with 1 ml of PBS, using the germicidal UV lamp. After exposure, a fresh growth medium with lenrispodun (UV + lenrispodun) or without lenrispodun (UV+) was added, and the samples were incubated for 4, 7, and 11 days. The third plate was used as a control without UV irradiation. RNA was harvested from each well, and cDNA was prepared from 5 μg of total RNA to quantify expression of the PDE1A, PDE1C genes, and senescence markers *p16* and *p21*. The effect of PDE1 inhibition was tested on the abovementioned genes in n = 6–10 samples from 3 independent duplicate experiments.

### Quantitative Real-Time PCR

The RNeasy Mini Kit (Qiagen) was used to isolate total RNA from the abdominal aorta of SMC-KO and SMC-LM mice. The concentration and quality of RNA samples were determined with a NanoDrop spectrophotometer (Isogen Life Science, IJsselstein, Netherlands). To prepare cDNA, RNA was reverse transcribed by the use of Quantitect Rev. Transcription Kit (Qiagen) according to the manufacturer’s protocol and then was amplified by quantitative real-time PCR on a QuantStudio 7 Flex Real-time PCR system (Applied Biosystems). All PCR measurements were performed in duplicate with SYBR Green PCR Master Mix (Applied Biosystems). PCR cycling conditions were 50°C for 2 min, then 95°C for 10 min, followed by 40 cycles of 95°C for 15 s and 60°C for 1 min β-actin and HPRT-1 were used as house-keeping genes in animal experiment. HPRT-1 and B2M were used as house-keeping genes in cell culture study. The expression values were corrected for expression levels of the two abovementioned house-keeping genes. The relative amount of genomic DNA in DNA samples was determined as follows: Relative Quantification (RQ) = 2 (-ΔΔCt). The primer pairs are provided in Table 1.

**TABLE 1:** qRT-PCR Primers.

Gene	Forward Primer	Reverse Primer
Pde1a^a^	5′ TCA​GAC​GAG​GTC​GGA​CGT​TGC 3′	5′ GGC​TGC​GCT​GAC​GTG​GTG​AT 3′
Pde1c^a^	5′ TGA​GAA​GCC​CAG​GTT​CAA​GAG 3′	5′ TCG​ATT​ACA​GCC​GGT​GGA​TAG 3′
Pde3a^a^	5′ ATA​CCT​GCT​CGG​ACT​CTG​AGG​A 3′	5′ TGG​CAG​AGG​TGG​TAG​TTG​TCC​A 3′
Pde5a^a^	5′ TGC​TTG​CAG​CTG​TAT​GAG​GCC​C 3′	5′ CGC​GAG​GGC​CTG​CCA​TTT​CT 3′
p16^a^	5′ CGC​TCT​GGC​TTT​CGT​GAA​CA 3′	5′ GTT​GCC​CAT​CAT​CAT​CAC​CTG​G 3′
p21^a^	5′ GTC​AGG​CTG​GTC​TGC​CTC​CG 3′	5′ CGG​TCC​CGT​GGA​CAG​TGA​GCA​G 3′
PDE1A^b^	5′ CCT​ATG​TGG​CAA​GCA​GCT​CA 3′	5′ CCC​ATC​ACT​CAT​GGA​GCC​TT 3′
PDE1C^b^	5′ GCA​GCC​AGA​AGC​CAT​TGA​AA 3′	5′ GGA​GTG​ACA​TTG​TCC​AGC​GA 3′
p16^b^	5′ TCGCGATGTCGCACGGTA 3′	5′ ATC​GGG​GAT​GTC​TGA​GGG​AC
p21^b^	5′ CCA​GCA​TGA​CAG​ATT​TCT​ACC​AC 3′	5′ CTT​CCT​GTG​GGC​GGA​TTA​GG 3′
β-actin^a^	5′ TTC​TTG​GGT​ATG​GAA​TCC​TGT​GG 3′	5′ GTC​TTT​ACG​GAT​GTC​AAC​GTC​AC 3′
HPRT-1^a^	5′ CCT​AAG​ATG​AGC​GCA​AGT​TGA​A 3′	5′ CCA​CAG​GAC​TAG​AAC​ACC​TGC​TAA 3′
B2M^b^	5′ CTC​CGT​GGC​CTT​AGC​TGT​G 3′	5′ TTT​GGA​GTA​CGC​TGG​ATA​GCC​T 3´
HPRT-1^b^	5′ TGA​CAC​TGG​CAA​AAC​AAT​GCA 3′	5′ GGT​CCT​TTT​CAC​CAG​CAA​GCT 3′

^a^Mice.

b Human.

### Vasodilator Function (*in vivo*)

To assess *in vivo* hindleg vasodilator function, a Laser Doppler perfusion imaging system (Perimed, PeriScan PIM 3 System) was used, as described before (Durik et al., 2012; Bautista Niño et al., 2015). In short, mice were anesthetized by 2.8% isoflurane/O^2^ ventilation (Penlon, Sigma Delta vaporizer), and the body temperature was kept at 36.4–37.0°C by means of a heating pad with rectal temperature probe feedback during the measurement. The hindleg, while kept in a fixed position, was occluded for 2 min with a tourniquet. After the release of the tourniquet, reactive hyperemia was measured for 10 min. Results were expressed as the area under the response curve and the maximum response.

### Sacrifice, Blood Collections, and Tissue Harvesting

At the age of 22 weeks, the animals were sacrificed by cardiac puncture and lethal blood withdrawal from the vena cava under anesthesia. Blood was centrifuged at 2,500 rpm at 4°C for 10 min, and plasma was obtained and stored at −80°C. The abdominal aorta was immediately frozen in liquid nitrogen and stored in −80°C for molecular analysis. The thoracic aorta and carotid artery were carefully isolated to use in organ bath experiments.

### Vascular Function Assessment (Ex Vivo)

The thoracic aorta was isolated and cleaned in cold oxygenated (with 95% O_2_ and 5% CO_2_) Krebs-Henseleit buffer solution (in mmol/L: NaCl 118, KCl 4.7, CaCl_2_ 2.5, MgSO_4_ 1.2, KH_2_PO_4_ 1.2, NaHCO_3_ 25 and glucose 8.3; pH 7.4) for *ex vivo* wire myography experiments. Vessel segments of 2-mm length were mounted in 6-ml chambers of wire myography device (Danish Myograph Technology, Aarhus, Denmark). To normalize the tension, the vessels were stretched in steps until 90% of the estimated diameter at which the effective transmural pressure of 100 mmHg was reached. After the normalizing procedure, the maximum contractile responses were measured using 100 mmol/L KCl. After 4 washing steps with a 5-min interval for each step, 30 nmol/L U46619 was applied to preconstrict the vessel segments and evaluate the relaxation concentration-response curves (CRCs) to ACh and SNP (respectively). ACh, by activation of endothelium, leads to vasodilation and SNP, activates the vascular smooth muscle cells directly, by release of NO. L-NAME 100 μmol/L, TRAM-34 10 μmol/L, and apamin 100 nmol/L were given 15 min before U46619 preconstriction, to investigate the involvement of nitric oxide and endothelium-dependent hyperpolarization (EDH) pathway in the relaxation responses. Lenrispodun 100 nmol/L was added to the organ baths 15 min before preconstriction to evaluate the acute effects of PDE1 inhibition.

### Mechanical Properties of the Vascular Wall

The carotid arteries explanted from 22-week-old SMC-KO and SMC-LM animals were isolated and cleaned from all the fat tissue. The carotid arteries were mounted in pressure myograph (Danish Myograph Technology, Aarhus, Denmark) in calcium-free buffer (in mmol/L: NaCl 120, KCl 5.9, EGTA 2, MgCl_2_ 3.6, NaH_2_PO_4_ 1.2, glucose 11.4, NaHCO_3_ 26.3; pH 7.4) to assess the passive properties of the vessel wall. The intraluminal pressure of the vessel was increased stepwise by 3-min, 10 mm Hg steps, starting from 10 mm Hg and ending at 120 mm Hg. At the end of each step, the vessels were allowed to equilibrate, and then the lumen diameter and wall thickness of the vessel were measured.

### Data Analysis and Statistics

Relaxation to ACh and SNP are expressed relative to the contraction produced by 30 nmol/L U46619, which were set at 100% in each individual aortic ring. Data are shown as the percentage of relaxation, expressed as the mean ± S.E.M. The number of each individual experiment is shown for each of the rings. Statistical comparisons of the different parameters were performed using IBM SPSS statistics (IBM Corporation, version 25) and GraphPad Prism (GraphPad Software Inc. version 8.0.1; San Diego, CA). Data were analyzed using unpaired two-sided *t*-test, one-way ANOVA followed by Dunnett’s post-hoc test, two-way ANOVA followed by Bonferroni’s post hoc test, and general linear model for repeated measurements (GLM-RM), as indicated in the figures and text for each case. *p* values less than 0.05 were considered significant.

## Results

### Diminished Endothelium-dependent and–Independent Relaxation in the Aorta and Mesenteric Arteries in Smooth Muscle Cell-specific Ercc1 KO Mice

**FIGURE 1:**
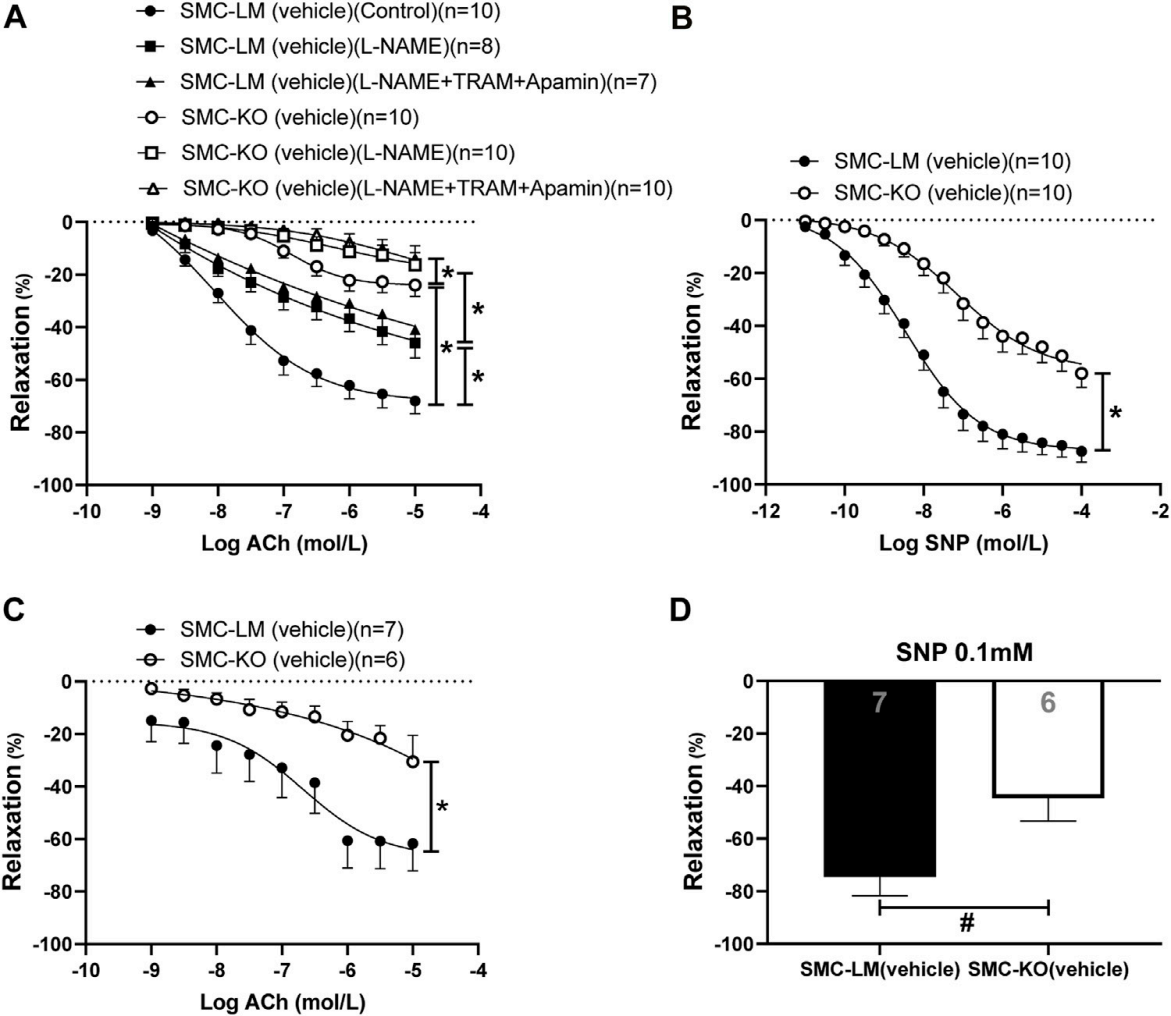
Endothelium-dependent and–independent responses and the contribution of NO and EDH pathways in isolated aortic rings **(A,B)** and the endothelium-dependent and–independent responses in mesenteric **(C,D)** rings from SMC-KO mice (open circles, bar) versus rings from SMC-LM (filled circles, bar) measured *ex vivo* in small wire organ baths. Relaxations are calculated relative to the contraction produced by U46619 30 mmol/L in each ring, which are set at 100%. Values are expressed as means ± SEM; n, number of mice; *, *p* < 0.05, GLM for repeated measures. #, *p* < 0.05, two-sided *t*-test.

The endothelium-dependent (ED) and -independent (EI) responses were significantly diminished in both conduit (aorta; Figures 1A,B) and resistance (mesenteric; Figures 1C,D) arteries of SMC-KO mice vs. SMC-LM at the age of 6 months. Blockade of eNOS by L-NAME decreased the relaxations in both SMC-KO and SMC-LM mice while the addition of the IK_Ca2+_ and SK_Ca2+_ channel blockers TRAM34 and apamin (which inhibit the EDH pathway) did not further reduce the relaxation on top of L-NAME, neither in SMC-KO mice nor in SMC-LM. Residual response after adding L-NAME (NO-independent relaxation) was also decreased in the aorta of SMC-KO mice compared to SMC-LM (Figure 1A).

### Effect of Acute vs. Chronic Treatment of Lenrispodun on Vasorelaxation in Organ Baths

**FIGURE 2:**
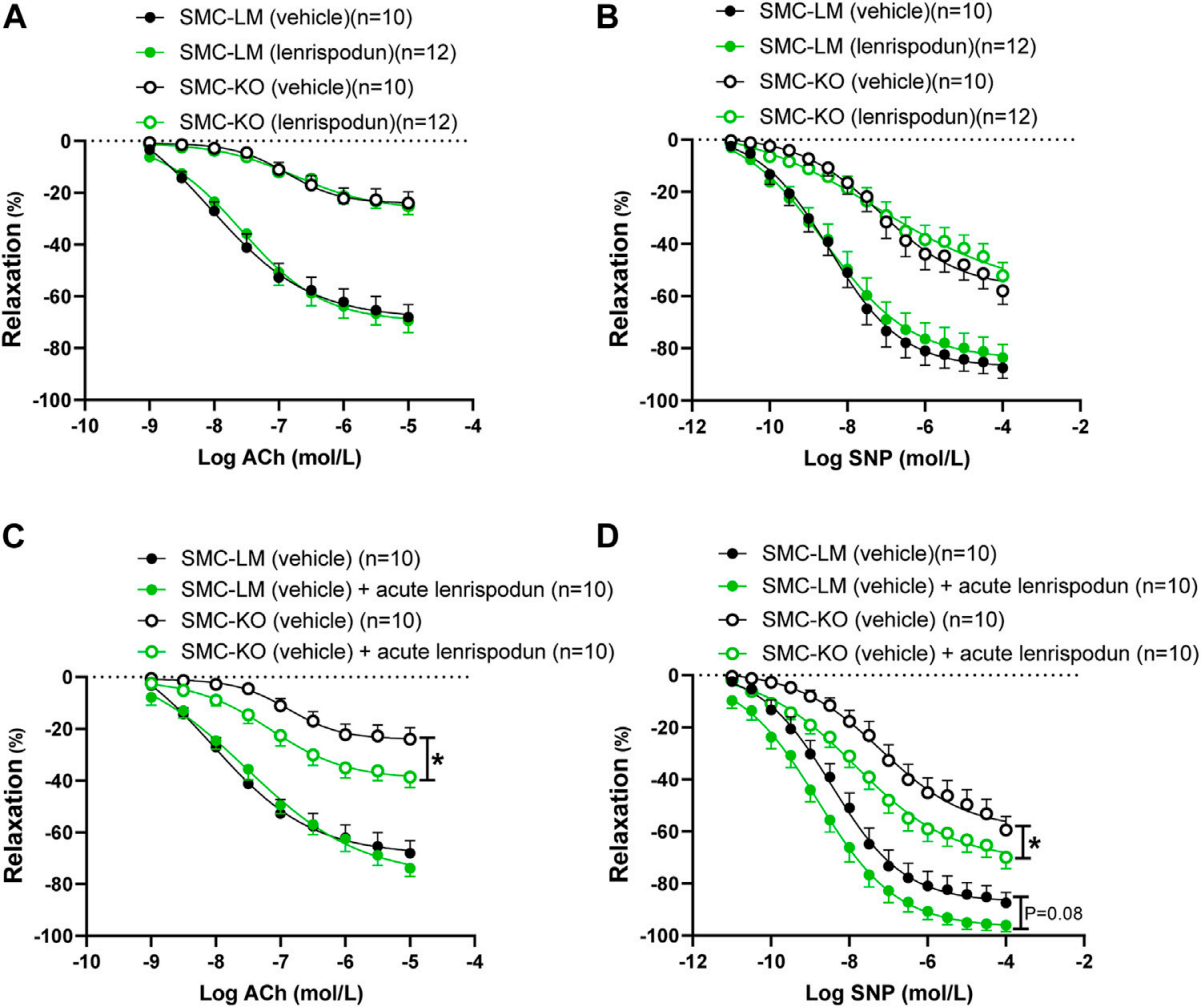
Effect of chronic **(A,B)** and acute **(C,D)** lenrispodun treatment on endothelium-dependent (ACh) and -independent (SNP) responses in isolated aortic rings from SMC-KO mice (open circles) versus rings from SMC-LM (filled circles), lenrispodun-treated samples are shown in green. Relaxations are calculated relative to the contraction produced by U46619 30 mmol/L in each ring, which are set at 100%. Values are expressed as means ± SEM; n, number of mice; *, *p* < 0.05, GLM for repeated measures.

After 12 weeks of treatment with lenrispodun, the ex vivo-measured endothelium-dependent and -independent responses remained unchanged in SMC-LM and SMC-KO animals, both in the aorta (Figures 2A,B) and the mesenteric artery (Supplemental Figure S1A, B). Also, NO–independent relaxation in the aorta of lenrispodun-treated SMC-LM and SMC-KO mice was similar to that in untreated mice (Supplemental Figure S1C). However, administration of lenrispodun acutely to the organ bath improved both ED and EI responses in SMC-KO mice (Figures 2C,D). A trend toward increased EI, but not ED, was also observed in SMC-LM animals in the presence of lenrispodun in the organ bath (*p* = 0.08) (Figures 2C,D).

### Effect of Lenrispodun on Vascular Stiffness

**FIGURE 3:**
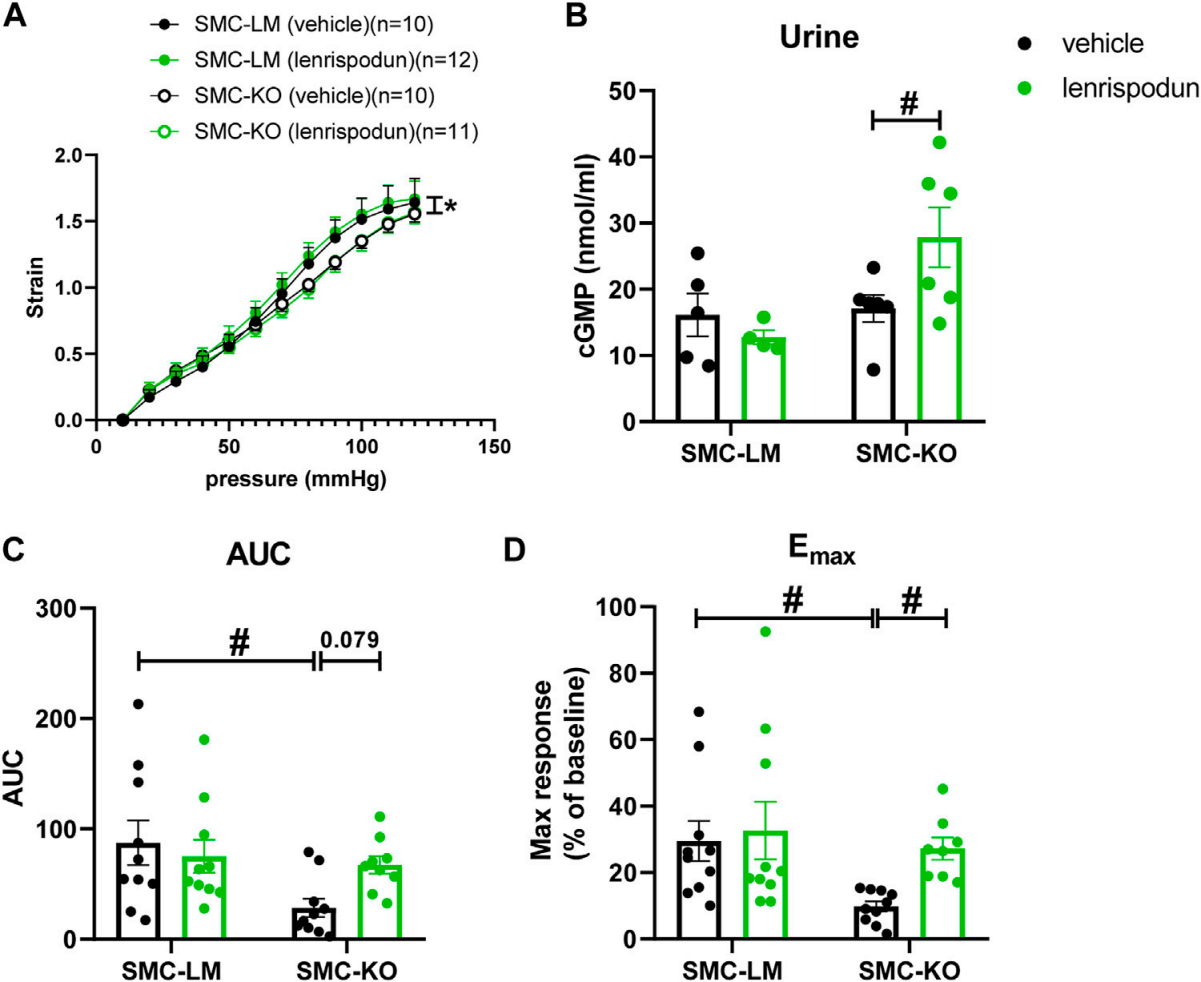
The *ex vivo* stiffness **(A)** in carotid artery segments of SMC-KO mice versus their SMC-LM. Lenrispodun-treated samples are shown in green. Compliance was measured by pressure myography technique and plotted as intraluminal pressure vs. strain. The cGMP content **(B)** in the urine of SMC-KO mice versus their SMC-LM, lenrispodun-treated samples are shown in green. *In vivo* cutaneous microvascular reactive hyperemia in SMC-KO mice versus their SMC-LM measured with Laser Doppler, expressed as **(C)** area under the curve (AUC) and **(D)** maximum response (Emax); *, *p* < 0.05, GLM for repeated measures; #, *p* < 0.05, two-way ANOVA followed by Bonferroni’s post hoc test.

Isolated carotid artery segments from SMC-KO showed less compliance to increasing intraluminal pressure than SMC-LM segments at the age of 6 months (Figure 3A). Chronic lenrispodun did not affect this outcome (Figure 3A).

### cGMP Content in the Urine

To assess the effects of PDE1 inhibition on cGMP concentration, the level of cGMP in the urine was measured. No difference was observed between untreated SMC-KO and SMC-LM animals. However, lenrispodun was able to increase the level of cGMP selectively in SMC-KO treated mice (Figure 3B).

### Effect of Lenrispodun on Microvascular Blood Flow *in vivo*

To evaluate the vascular function of smaller arteries *in vivo*, a situation in which lenrispodun is still present, the superficial blood flow of skin resistance vasculature was measured with Laser Doppler reactive hyperemia tests. Reactive hyperemia was not significantly decreased at 3 months of age (Supplemental Figure S1D, E). At 6 months of age, reactive hyperemia in vehicle-treated SMC-KO is lower than in SMC-LM littermates (Figures 3C,D). No difference is observed in chronic lenrispodun-treated SMC-KO mice compared to both vehicle-treated and lenrispodun-treated SMC-LT. Lenrispodun treatment improved the reactive hyperemia maximum response selectively in SMC-KO mice, and a significant trend toward improvement of the area under the curve (AUC) is observed (*p* = 0.079). This observation is in line with the acute effect on ACh and SNP responses selectively in SMC-KO in Figure 2C.

### The Plasma Inflammatory Status After PDE1 Inhibition

**FIGURE 4:**
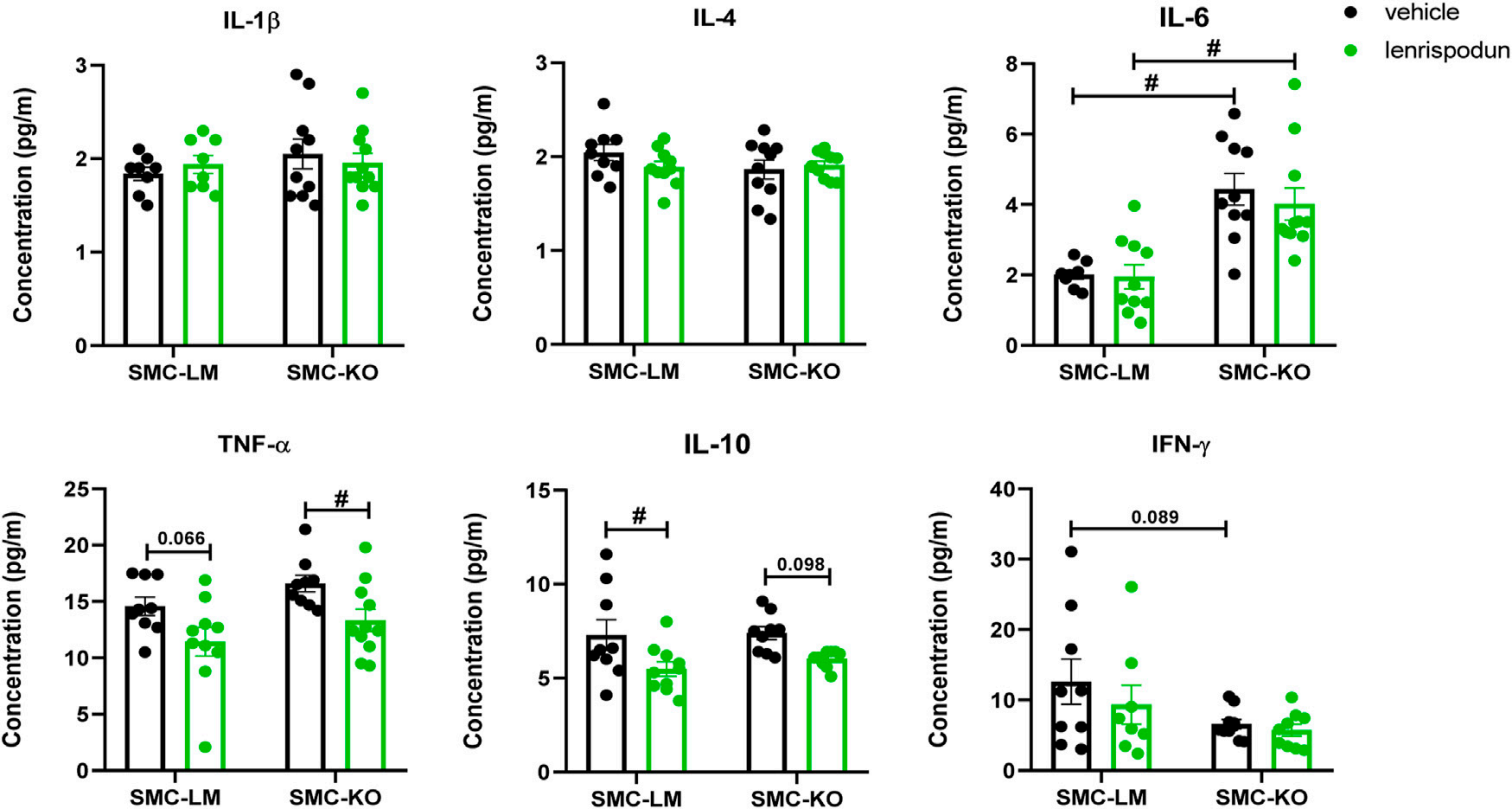
The level of inflammatory cytokines in the plasma of SMC-KO mice versus their SMC-LM, lenrispodun-treated samples are shown in green. Values are expressed as means ± SEM; n = 4–11; #, *p* < 0.05, two-way ANOVA followed by Bonferroni’s post hoc test.

Plasma IL-6 was increased in vehicle-treated SMC-KO vs. SMC-LM, while no differences were observed in IL-1β, IL-4, TNF-α, IL-10 and IFN-γ. The concentration of TNF-α was significantly decreased by lenrispodun treatment in SMC-KO mice, and a similar trend was observed in SMC-LM animals (*p* = 0.066). The level of IL-10 was reduced in lenrispodun-treated SMC-LT mice, and a similar trend was observed in SMC-KO mice (*p* = 0.098) (Figure 4).

### PDEs, MRPs and Senescence Markers Expression

**FIGURE 5:**
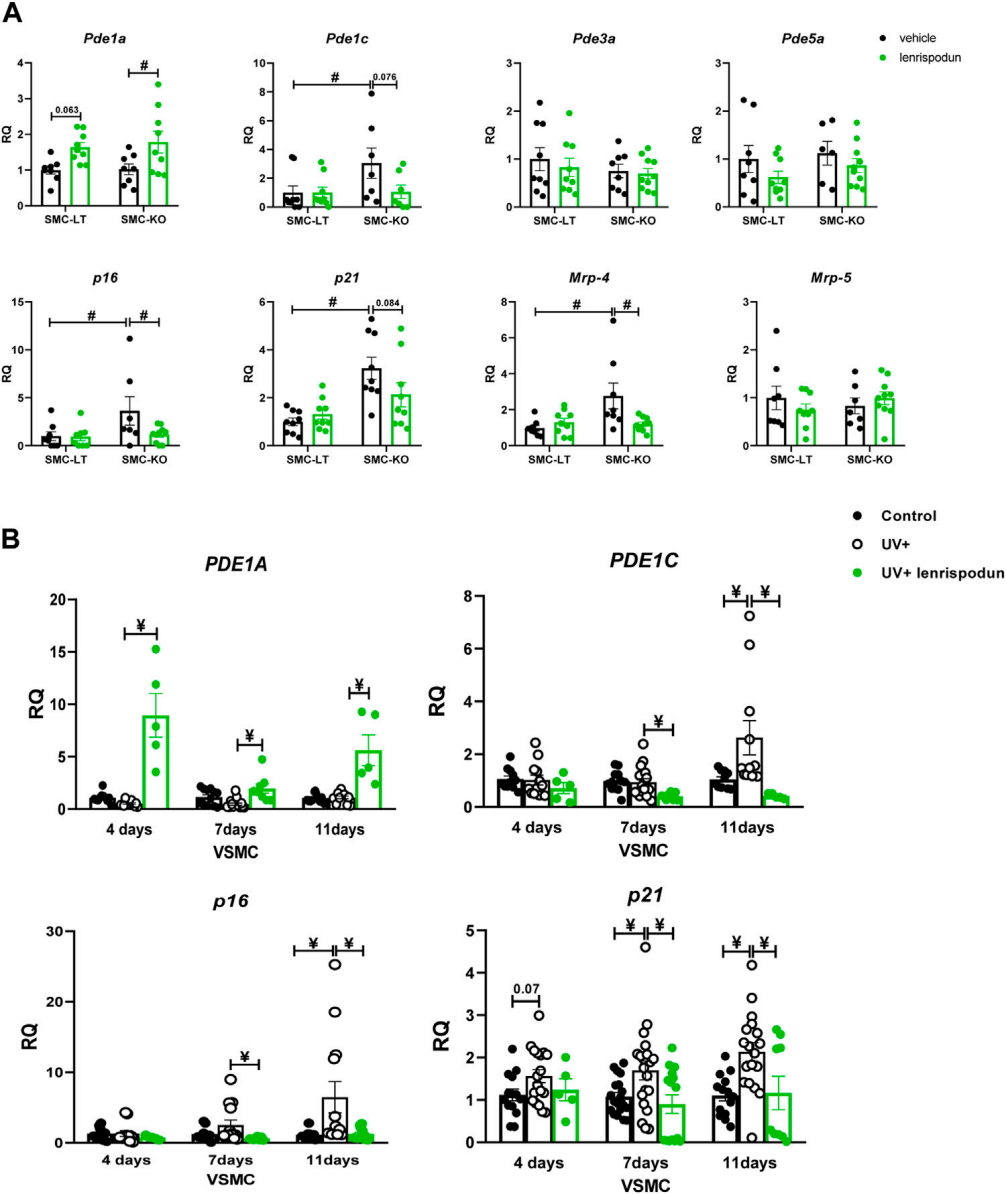
The mRNA expression of PDEs, MRPs and senescence markers in the abdominal aorta of SMC-KO mice versus their SMC-LM **(A)**, the mRNA expression of PDEs and senescence markers in cultured human vascular smooth muscle cells **(B)**, measured by RT-qPCR technique, lenrispodun-treated samples are shown in green. Values are expressed as means ± SEM; n = 6–8; #, *p* < 0.05, two-way ANOVA followed by Bonferroni’s post hoc test; ¥, *p* < 0.05, one-way ANOVA followed by Dunnett’s post-hoc test (all the comparisons were made compare to UV+ group).

The mRNA expression of PDEs that are known to be involved in cGMP regulation in VSMC was measured in the abdominal aorta (Figure 5A). The expression of *Pde1a* was increased in SMC-KO mice by lenrispodun treatment, and a similar trend was observed in SMC-LT mice (*p* = 0.063). *Pde1c* expression was elevated in vehicle-treated SMC-KO mice compared to SMC-LT, and the lenrispodun treatment reversed this difference. The expression of *Pde3a* and *Pde5a* was the same in vehicle-treated SMC-KO and SMC-LT and remained unchanged after the treatment. We previously found no contribution of soluble guanylyl cyclase or protein kinase G to Ercc1 mutant mouse cGMP responses (Durik et al., 2012; Bautista Niño et al., 2015). To investigate other possible regulators of cGMP levels, we evaluated the expression of multidrug resistance-associated protein (MRP) 4 and 5. These were recently identified as cGMP channels. *Mrp-4* mRNA was increased in aorta of SMC-KO. This was normalized by lenrispodun (Figure 5). *Mrp-5* was unaffected by genotype and treatment. The senescence-related markers, *p16* and *p21*, were elevated in SMC-KO vs. SMC-LM. Lenrispodun decreased the level of *p16* selectively in SMC-KO mice, and a similar trend was observed for *p21* (*p* = 0.084).

To confirm the local VSMC effect of DNA damage, independently from ERCC1 deletion, on *PDE1A*, *PDE1C,* and senescence markers expression, an *in-vitro* human vascular smooth muscle cell model of UV irradiation was used (Figure 5B). The level of *PDE1A* was unchanged by UV irradiation but increased in lenrispodun-treated, irradiated cells. *PDE1C* expression was increased 11 days after the irradiation, whereas it was reduced by lenrispodun treatment. Both *p16* and *p21* showed a time-dependent increase after the irradiation compared to control cells, and lenrispodun treatment prevented this. IL-6 mRNA was induced at 6 h after UV irradiation. After a rapid decrease at 12 and 24 h, it normalized again after 4 days (Supplementary Figure S2).

## Discussion

In this study, we employed a pharmacological approach to inhibit PDE1, both *in vitro* and *in vivo*, and to explore the role of PDE1 in VSMC-specific features of aging and vascular dysfunction. We here confirm that at the age of 22 weeks, SMC-KO mice display a pronounced vasodilator dysfunction in the isolated aorta and cutaneous microvasculature, and for the first time, establish that mesenteric arteries show a similar dysfunction. We found that PDE1 inhibition with lenrispodun can potentiate NO/cGMP signaling and improve vasodilation after acute administration. This improvement in endothelium-dependent and -independent responses is selective in mice with aged VSMC versus non-aged control mice. *Ex vivo*, these effects occurred acutely by adding the drug to the organ bath but were not observed after chronic treatment and subsequent mounting of the arterial tissue in the organ bath without the addition of the drug. *In vivo*, an enhanced microvascular response is seen in the presence of lenrispodun in association with upregulated cGMP levels in SMC-KO mice (confirmed in urine). The cGMP measurement is sensitive enough to pick up even less than 0.1 pmol/L concentrations, yet no increase was observed in SMC-LM. It is known that PDE1 does not appreciably increase cGMP levels in the blood in experiments with middle-aged humans or in cell culture homogenates. This is attributed to its function in nanodomains (Hashimoto et al., 2018; Gilotra et al., 2021). Since SMC-KO mice are strongly aged, our current findings support the hypothesis that PDE1 function drastically changes due to aging, even to the point that it unmasks a cGMP increase. Further, chronic lenrispodun treatment decreased TNF-α and IL-10 plasma levels. IL-6 was selectively increased in SMC-KO but not decreased by PDE1 inhibition, suggesting that cytokines are differentially affected by DNA damage in the SMC-KO mice. Senescence markers p16 and p21 were elevated in the VSMC and SMC-KO mice and these senescence markers were lowered by lenrispodun. Increases in expression of *Pde1c* and senescence markers were associated with aging in both animal models and cell culture experiments, while IL-6 is only increased by DNA damage but not related to senescence in VSMC. Lenrispodun can ameliorate the increases in senescence markers and *Pde1c*. However, in SMC-KO, the presence of the drug is required to restore endothelium-dependent and -independent vasodilator relaxations in the *ex-vivo* situation. With respect to the role of blood pressure, we have shown previously that SMC-KO are normotensive (Ataabadi et al., 2021). In addition, systolic and diastolic blood pressure lowering with Ang II type 1 receptor antagonist, losartan, did not improve vascular aging features in whole body Ercc1 KO mice model (Wu et al., 2017). Therefore, a role for blood pressure is highly unlikely and blood pressure monitoring was not applied in the current experiment.

The selective effect of lenrispodun in aged mice underlines our previous observation regarding the role of reduced PDE1 activity in aged vessels. This previous result was derived from experiments in *Ercc1* ^*Δ/-*^ mice, a whole-body ERCC1-defective model that shows accelerated aging in many organs (Dollé et al., 2011; Bautista Niño et al., 2015). Decreased vasodilation in *Ercc1* ^*Δ/-*^ mice was found to be derived from three mechanisms: decreased eNOS activity, increased cGMP metabolism, presumably by PDE1, and increased oxidative stress. The current finding in SMC-KO underlines the involvement of PDE1 in untreated mice. However, after chronic lenrispodun treatment, *Pde1c* has normalized, but *Pde1a* is increased. For SMC-LM, this is of no effect, but in SMC-KO arteries, vasodilation in organ baths is still decreased after treatment. This might be explained by the presence of oxidative stress involving NADPH oxidase 2 in SMC-KO, which creates stringent conditions for NO–cGMP signaling as it decreases NO bioavailability by its conversion to peroxynitrite (Ataabadi et al., 2020; Ataabadi et al., 2021). Indeed, the upregulation of *Pde1a* mRNA expression has previously been shown to act as a counter-mechanism and play a role in nitrate tolerance, thereby providing another feasible explanation of decreased cGMP responses (Kim et al., 2001). *Mrp-4* was increased in SMC-KO, which might contribute to decreased cGMP responsiveness as it transports the cyclic nucleotide to the extracellular space. This was restored to the level of SMC-LM by lenrispodun in SMC-KO mice while no effect was observed in SMC-LM, underlining the exclusiveness of PDE1 changes in aged aorta. Concludingly, various changes might determine the ultimate response in isolated aortic ring of lenrispodun-treated mice. For an optimal effect on vasodilations, the constant presence of this PDE1 inhibitor is needed.

In *Ercc1* ^*Δ/-*^ mice, the effect of chronic PDE1 inhibition was sustained after removal of the arterial tissue and subsequent measurement in the organ bath. This discrepancy might be explained by the predominant role and more advanced state of VSMC dysfunction in the aorta of SMC-KO (39% loss of response to 0.1 mM SNP) compared to *Ercc1* ^*Δ/-*^ mice (21% loss of response to 0.1 mM SNP (Golshiri et al., 2021)). The *Ercc1* ^*Δ/-*^ mutation preserved one allele that produces a partially active, mutant ERCC1 protein, whereas the presently used SMC-KO is a full gene knockout. Therefore, we cannot rule out that in SMC-KO, the severity of the DNA repair defect, and therefore of the aging process, has mitigated the chronic effect of lenrispodun treatment. An alternative explanation is that in *Ercc1* ^*Δ/-*^ mice, the development of vascular dysfunction and the effect of PDE1 inhibition is exerted through modulation of other cell types in addition to VSMC.

The involvement of other cell types than VSMC is also supported by the differential effect of lenrispodun on plasma cytokines in *Ercc1* ^*Δ/-*^ mice vs. SMC-KO. Cytokines IL-1β and TNF-α were increased in *Ercc1* ^*Δ/-*^ mice (Golshiri et al., 2021) and inhibited by lenrispodun. These cytokines were unchanged in SMC-KO, both by genotype and treatment, implicating a non-VSMC origin in *Ercc1* ^*Δ/-*^ mice. Accordingly, unlike the vasomotor effect of lenrispodun, the effect of this PDE1 inhibitor on cytokines is not accentuated in SMC-KO compared to SMC-LM. In contrast to TNF-α and IL-1β, IL-6 is increased in SMC-KO. The cell culture experiments in VSMC indicate that this is related to the acute response following DNA damage. In VSMC, IL-6 regulation is unrelated to PDE1, as it occurs before the increase of PDE1C and is not responsive to lenrispodun. In comparison, *Ercc1* ^*Δ/-*^ mice show a much greater increase of IL-6 than can be explained by VSMC alone and IL-6 increases are decreased after chronic lenrispodun treatment in this model (Khammy et al., 2017). Also, these findings suggest the involvement of cell types other than VSMC contribute to the effect of genotype and lenrispodun treatment in *Ercc1* ^*Δ/-*^ mice.

The increase of inflammatory cytokines, such as IL-6 and TNF-α, in aging has been linked to the cellular senescence-associated secretory phenotype (SASP) (Sanada et al., 2018). This potential link was confirmed for some organs of *Ercc1* ^*Δ/-*^ mice, namely liver, kidney, and lung (Yousefzadeh et al., 2021b). Our present results, however, show that the effect of DNA damage and PDE1 inhibition on cytokines is dissociated from senescence in VSMC, both *in vitro* and *in vivo*. This is in agreement with the growing awareness that SASP is cell type-specific (Basisty et al., 2020). Nevertheless, we here observe an effect on senescence markers *p16* and *p21*. In a model of abdominal aorta aneurysm, it was shown that PDE1C knockout attenuated aneurysm formation and lowered VSMC senescence involving *p21*. Allosteric activation of the senescence-regulating deacetylase Sirtuin-1 by cAMP, which is increased by PDE1 inhibition, was implicated (Zhang et al., 2021). Remarkably, we here demonstrate that PDE1C is gradually increased after DNA damage, to ultimately coincide with increased senescence, and that both of these changes can be blocked by lenrispodun treatment. Jointly, the data suggest that the PDE1C subtype is importantly involved in the regulation of senescence in VSMC, and that may not be related to altered cytokine expression that is typically associated with SASP.

In conclusion, we demonstrated that aging-induced by deletion of ERCC1 enzyme in SMC, determines the responsiveness to acute PDE1 inhibition in vascular dysfunction. For the effect of chronic intervention, the interplay between differential PDE1A and C expression and/or the state of progression of the aging process are potential determinants of drug efficacy. In conditions of strongly advanced aging, the continuous presence of PDE1 inhibitor might be required to optimally suppress the established detrimental pathogenic mechanisms that are at play. For the current clinical research and applications, this is pivotal. Long-term effects of (pharmaco) therapy on aging can currently not be tested in humans from youth onward, due to the lack of high-resolution general aging markers. Therapy will only be conducted after establishing the clinical result of a longstanding, progressed non-atherosclerotic disease. Typical examples of such diseases are vascular dementia, cardiac ischemia due to non-obstructive coronary artery disease, better known as INOCA, and heart failure with preserved ejection fraction. The future of PDE1 inhibitor may lie in these fields of application, for which currently no therapy exists.

## Data Availability

The original contributions presented in the study are included in the article/Supplementary Material, further inquiries can be directed to the corresponding author.
