# Supplementary material for: The Effects of Acute and Chronic Selective Phosphodiesterase 1 Inhibition on Smooth Muscle Cell-Associated Aging Features
Source: Front Pharmacol. 2022 Jan 31;12:818355. doi: 10.3389/fphar.2021.818355 (PMC8841451; doi:10.3389/fphar.2021.818355)
Supplement: Supplementary file 1 [file DataSheet1.pdf]

Legends:

Figure S1. Endothelium-dependent (A) and -independent (B) responses in isolated mesenteric rings from SMC-KO mice (open circles, bars) versus rings from SMC-LM (filled circles, bars), measured *ex vivo* in small wire organ baths. The effect of 12 weeks of treatment with lenrispodun given in drinking water is shown in green. The contribution of NO-independent pathway (L-NAME treatment) after chronic lenrispodun treatment is shown in panel C for aorta rings. *In vivo* cutaneous microvascular reactive hyperemia in SMC-KO mice versus their SMC-LM at the age of 3 month measured with laser Doppler, expressed as (D) area under the curve (AUC) and (E) maximum response (Emax). Relaxations are calculated relative to the contraction produced by U46619 in each ring, which is set at 100%. Values are expressed as means ± SEM; n, number of mice; *, $p < 0.05$; GLM for repeated measures; #, $p < 0.05$, two-way ANOVA followed by Bonferroni's post hoc test. In figures D and E, two-sided t-test was used.

Figure S2. The mRNA expression of IL-6 in cultured human vascular smooth muscle cells, measured by RT-qPCR technique. Values are expressed as means ± SEM; n=4-7; #, $p < 0.05$, two-sided t-test.

Figure S1

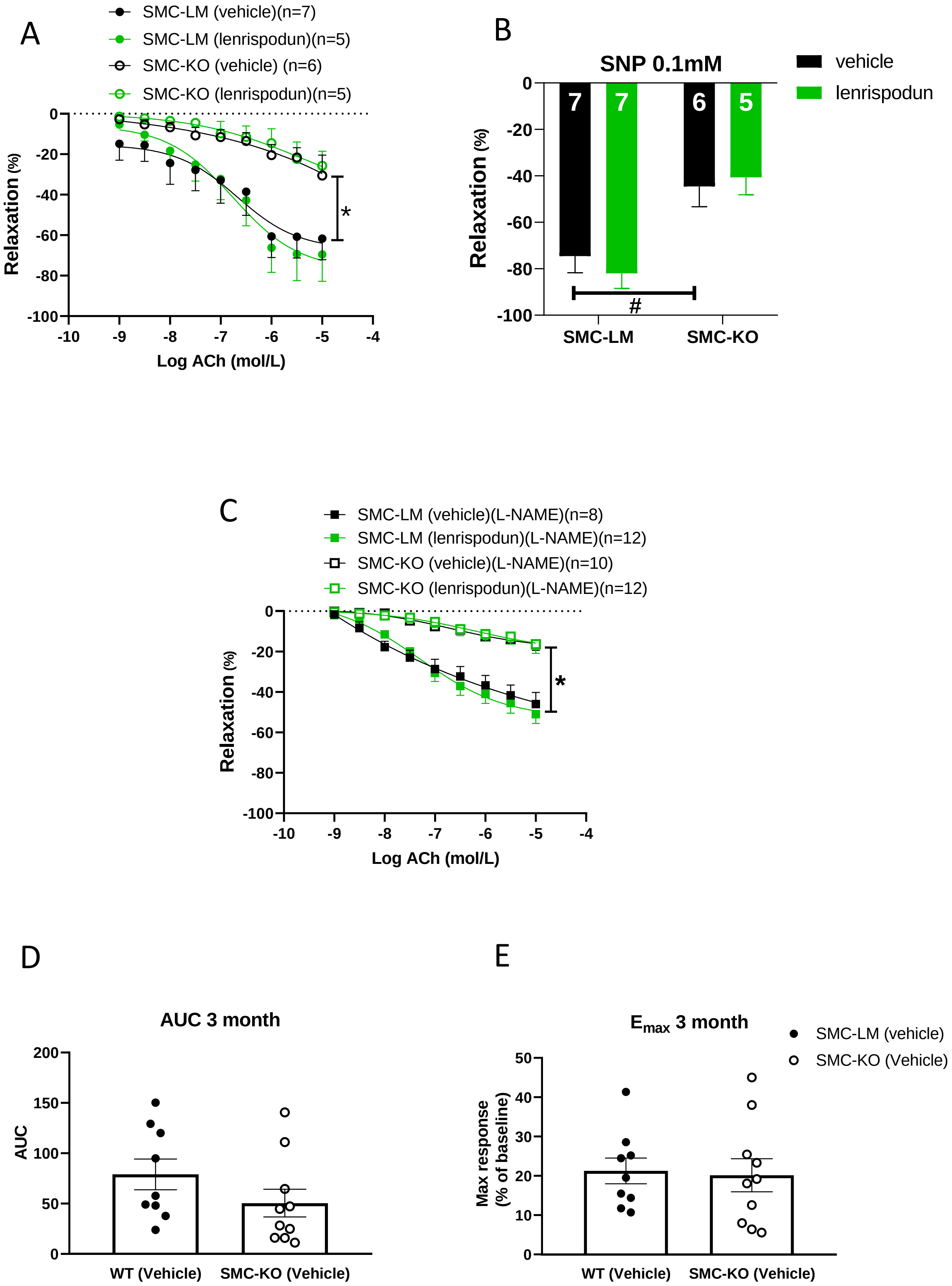

A
SMC-LM (vehicle)(n=7)
SMC-LM (lenrispodun)(n=5)
SMC-KO (vehicle) (n=6)
SMC-KO (lenrispodun)(n=5)
Relaxation (%)
Log ACh (mol/L)
*
B
SNP 0.1mM
vehicle
lenrispodun
Relaxation (%)
7
7
6
5
#
SMC-LM
SMC-KO
C
SMC-LM (vehicle)(L-NAME)(n=8)
SMC-LM (lenrispodun)(L-NAME)(n=12)
SMC-KO (vehicle)(L-NAME)(n=10)
SMC-KO (lenrispodun)(L-NAME)(n=12)
Relaxation (%)
Log ACh (mol/L)
*
D
AUC 3 month
AUC
WT (Vehicle)
SMC-KO (Vehicle)
E
E_{max} 3 month
SMC-LM (vehicle)
SMC-KO (Vehicle)
Max response
(% of baseline)
WT (Vehicle)
SMC-KO (Vehicle)

Figure S2

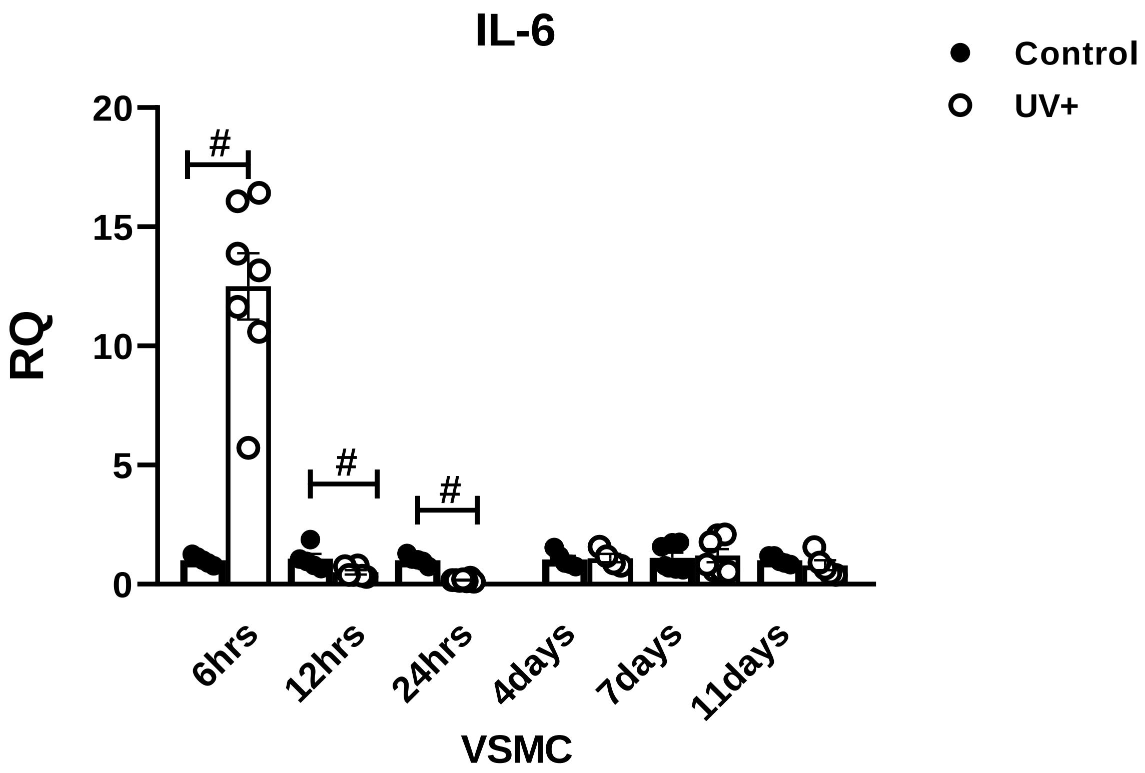

IL-6
Control
UV+
RQ
20
15
10
5
0
#
#
#
6hrs
12hrs
24hrs
4days
7days
11days
VSMC
